# START – introducing a novel assessment of consultant readiness in Paediatrics: the entry not the exit

**DOI:** 10.15694/mep.2020.000095.1

**Published:** 2020-05-12

**Authors:** Ashley Reece, Lucy Foard

**Affiliations:** 1 Royal College of Paediatrics and Child Health; 2 Royal College of Paediatrics and Child Health

**Keywords:** Consultant preparation, Consultant readiness, Training, Paediatrics, CCT, Assessment, OSCE, Postgraduate, Examinations, Transition

## Abstract

This article was migrated. The article was not marked as recommended.

The Royal College of Paediatrics and Child Health (RCPCH) developed a new end-of-training assessment held for the first time in 2012, known as START, the Specialty Trainee Assessment of Readiness for Tenure as a consultant. It is a novel, formative, multi-scenario, OSCE-style, out-of-workplace assessment using unseen scenarios with generic, external assessors undertaken in the trainees’ penultimate training year. This paper describes the introduction and structure of this formative assessment. While many other colleges have summative exit exams the inception of this assessment was designed to be formative, providing feedback on consultant-readiness skills, and not a high-stakes hurdle towards the end of training, It was developed from the College’s examinations question-setting group and following two pilots in 2009 and 2010, the assessment evolved and the first live diet was held in November 2012.

## Introduction

### Background

This paper describes the Royal College of Paediatrics and Child Health’s (RCPCH), assessment towards the end of specialist paediatric training, known as START, an acronym of the Specialty Trainee Assessment of Readiness for Tenure as a consultant.

### Development of the assessment

In 2007 the RCPCH reviewed their requirements for completion of paediatric training. Unlike some other colleges who hold high-stakes, pass/fail, summative assessments (computer-based knowledge, situational judgement tests, or formal clinical viva voce examinations) there was no ‘exit’ examination in paediatric training. At that time Paediatricians leading training did not want to make trainees take a high-stakes hurdle to become a paediatric consultant after eight years of specialist training with a risk they might not pass a one-off summative assessment. In that spirit, a formative assessment in the penultimate training year was proposed. The aim was to assess trainees in their final training period (known as ‘level 3’ training) in different scenarios across multiple domains in the style of an Objective Structured Clinical Examination (OSCE) ( Harden and Gleeson, 1979). A multi-station, formative assessment taken in the seventh Specialty Training year, called the ‘sT7 Assessment’ (ST7A), was devised. Twelve eight-minute stations, mainly using a structured oral as a basis for a directed discussion covering a series of predetermined consultant-orientated scenarios, were assessed by consultants trained to be assessors in this assessment, judging key competencies against the expected agreed standard of a newly appointed consultant. Two pilots ran in 2009 and 2010 and data generated from these, and from questionnaires to trainees and assessors, reported positive responses about the assessment with trainees feeling they had not been tested in these areas in other ways in training and welcoming the opportunity to ‘think like a consultant’ in preparation for consultant posts. Assessors also viewed the assessment favourably too ( McGraw, 2010). Following these successful pilots, the General Medical Council (GMC) gave the RCPCH a mandate to include the assessment within the College’s assessment strategy and the name of the assessment was changed to START. Since the assessment is formative, a different lexicon of terminology was developed to use when discussing START as opposed to the summative college membership exams. This is detailed in table 1.

**Table 1.** Terminology used in the START assessment compared to exams

Membership examinations	START Assessment
Pass/Fail	Meeting competency/standard
Standard setting	Benchmarking
Station	Scenario
Examiner	Assessor
Candidate	Trainee
Mark sheet	Feedback form
Senior Examiner	Supporting Assessor

## Details of the assessment

### Scenarios and circuit

The stations, known as ‘scenarios’, cover the following areas: Case-based discussion, Ward round and handover, Logistics and organisation, Safeguarding children, Critical appraisal of literature, Safe prescribing, Ethics, Consent and law, Teaching and Conflict and risk management. At the time of writing, each trainee completes 12 scenarios; 6 speciality-specific and 6 general paediatric.

START scenarios are written around real-life clinical, managerial and logistical episodes which the trainee discusses with the assessor. The trainees are given a vignette and have four minutes to think through their approach. For the critical appraisal and prescribing they have a 45-minute block to prepare set tasks. In an OSCE format the trainees move through the 12 scenarios, holding a discussion with an assessor for 8 minutes in each one. Knowledge, while tacit, is not the sole determinant of the assessment of performance. Some of the scenarios allow the trainees to demonstrate higher order skills from Miller’s pyramid ( Miller, 1990; Cheek, 2010), for example, writing a prescription, doing a critical appraisal and the real-time micro-teach to medical students which evolved after the early diets ( Reece and Fertleman, 2015).

### Feedback on performance

Trainees are graded on their intra-scenario performance during a professional conversation, in the style of Schön (1983), who believed that exploring specific experiences would help learners acquire ‘knowing-in-action’ if coached by expert practitioners. Assessors are generic and not specialist and grade trainees’ performance in the scenario across six domains mapping to the GMC’s Good Medical Practice as ‘further development required’, ‘performed at expected standard’ or ‘performed well above the expected standard’ ( General Medical Council, 2013), recorded as the rating for each item, giving an item rating. As well as this, a global rating of ‘development needed’, ‘meets competence’, ‘above competence’ or ‘significant concern’ is given. The ‘significant concern’ identifies very sub-standard performance in that scenario requiring specific attention. Appendix 1 shows the benchmarking grid structure. Each assessor types feedback on the performance in each scenario directly into an electronic repository during the assessment. This is subsequently reviewed and released to trainees about 6 weeks after the assessment following grammatical, spelling and sense check. All ‘significant concern’ ratings are scrutinised by senior assessors both during the assessment and at a review meeting after the assessment (the START Executive Committee). The feedback is then available to the trainee and their educational supervisor and informs a Personal Development Plan supporting targeted learning and training opportunities in the trainee’s final training year, documented and evidenced in their learning e-portfolio. So, the value of START hinges on valuable feedback and support from the trainee’s educational supervisor. A document has been produced to enable educational supervisors to support trainees in making the most of the feedback. Access to relevant local learning opportunities vary locally within Deaneries.

Performance and feedback at START are not the sole determinants of progression at Assessment Review of Competence Progression (ARCP) but one of the assessment tools alongside workplace-based assessments, multi-source feedback, reflection, trainers’ reports and ePortfolio evidence. START is not used to inform a consultant appointment interview panel as it is not designed for that purpose. After each sitting the RCPCH surveys trainees and assessors.

After the first five sittings between November 2012 and October 2014, 509 paediatric trainees had performed the assessment; 273 responded to a survey (response rate 54%). From 181 assessors, 112 responded (response rate 62%). Responses showed acceptability by trainees and assessors ( Reece *et al*., 2015).

### Assessors

Assessors are Consultant Paediatricians and Fellows of the RCPCH who have applied to assess START; many are involved in assessment and have a particular interest in education and training. They attend a single day of training and are given a refresher on the day of the assessment itself. During the assessment, over later diets, they have been given peer-review of their performance in-assessment by supporting assessors. This is reviewed and returned to the individual consultant assessor to use in their own education portfolio. 

## Results

### Performance data from all diets to date

#### Number of trainees and assessors

The numbers of trainees and assessors for each assessment are indicated in table 2 below. Many assessors have assessed across many assessments. The sitting in November 2017 was a two-circuit assessment held outside London. This was an extra assessment was held that year to ensure all the trainees in approaching their final year could access an assessment.

**Table 2.** Number of trainees and assessors for START

Date	Trainees	Assessors
Nov-12	59	37
Mar-13	88	37
Oct-13	98	37
Mar-14	123	37
Oct-14	141	38
Apr-15	145	36
Oct-15	126	38
Apr-16	137	39
Oct-16	140	42
Mar-17	144	42
Oct-17	140	42
Nov-17	47	25
Apr-18	141	45
**Total**	**1529**	**495**

Table 3 details the speciality mix of the START sessions to 2018.

**Table 3.** The mix of trainees from different specialities over each assessment

Paediatric Sub-specialities	Assessment Dates													Total number of Trainees
	2012	2013		2014		2015		2016		2017			2018	
	Nov	Mar	Oct	Mar	Oct	Apr	Oct	Apr	Oct	Mar	Oct	Nov	Apr	
**Allergy**				1	1	3	1			1	1		1	**9**
**Child Mental Health**													1	**1**
**Community Child Health**	5	1	4	11	10	13	15	16	11	12		20	13	**131**
**Diabetes and Endocrinology**						1		3	2	1	3			**10**
**Emergency Medicine**	7	3	1											**11**
**General Paediatrics**	41	69	83	84	99	78	77	85	83	89	98	19	92	**997**
**Immunology and Infectious Disease**	1			1			1			1	3			**7**
**Metabolic Medicine**					1		1			1			1	**4**
**Neonatal Medicine**	2	4	4	9	15	21	6	9	13	11		8	10	**112**
**Paediatric Neurology**		2												**2**
**Paediatric Emergency Medicine**				2	2	3	7	7	4	2	10		6	**43**
**Paediatric Gastroenterology, Hepatology & Nutrition**		2	1	3	2	3		3	7	3	3			**27**
**Paediatric Intensive Care Medicine**		1	3	1	3	6	8	1	7	5	8		8	**51**
**Paediatric Nephrology**		1		2		2	3		2	4	3		3	**20**
**Paediatric Neurodisability**	2	5		3	2	2	3	1	1	3	2			**24**
**Paediatric Neurology**			1		3	2	4	2	1	5	2		1	**21**
**Paediatric Oncology**						5		2	3	1	2		2	**15**
**Paediatric Respiratory**	1		1	3	1	4		3	6	3	2		2	**26**
**Paediatric Rheumatology**				2	1	2		3		1	1			**10**
**Palliative Medicine**				1	1			2		1	2		1	**8**
**Totals**	**59**	**88**	**98**	**123**	**141**	**145**	**126**	**137**	**140**	**144**	**140**	**47**	**141**	**1529**

#### Data Analysis

A psychometrician reviews all the data and produces a report after each diet which is reviewed by the START Executive Committee. For the most part the report presents stacked bar charts showing percentages of the descriptor categories. While it is important not to regard the descriptors on a numerical scale, to make some statistical sense of the assessment, the use of numerical scales to present global ratings, average global ratings, internal consistencies and assessor error bars were used. The global ratings for all scenarios were calculated using the following numbers assigned to the scale from the benchmarking standard scales: Significant concerns = 1, Development needed = 2, Meets competency = 3 and Above competence = 4. Item scores for each domain were calculated similarly: Further development required = 1, Performed at expected standard = 2 and Well above standard = 3.

Cronbach’s alphas are calculated to provide a measure of the internal consistency of START; this is a measure of the reliability of the assessment and that the scenarios measure the same overarching construct. Separate alpha values were calculated for the global ratings and item ratings. It is possible to determine the aggregated score of the six competency ratings per trainee per assessment. Alpha values are stable for the whole cohort for the 12 scenarios across diets, with means of α = 0.70 for the global ratings and α = 0.72 for the item ratings. Table 4 details the scores for the START sessions to date.

**Table 4.** Cronbach’s alpha for the whole cohort (number of trainees given in brackets) for global and item ratings having converted to scores as indicated above

Diet date	Global ratings for 12 scenarios (number of trainees)	Item ratings for 12 scenarios
Nov-12	0.785 (58)	0.777
Mar-13	0.744 (88)	0.714
Oct-13	0.692 (98)	0.745
Mar-14	0.701 (123)	0.690
Oct-14	0.708 (141)	0.744
Apr-15	0.676 (145)	0.704
Oct-15	0.684 (126)	0.707
Apr-16	0.690 (137)	0.707
Oct-16	0.647 (140)	0.707
Mar-17	0.725 (144)	0.726
Oct-17	0.656 (140)	0.685
Nov-17	0.694 (47) ^*^	0.691
Apr-18	0.713 (141)	0.710

* extra cohort, therefore smaller n and only 1 day.

#### Examples of formative feedback

The trainees receive feedback that is not numerical in nature; they receive descriptors for global and item ratings as well as written feedback.

An exemplar of the formative feedback provided to trainees is included in appendix 2.

## Discussion

Over 12 diets the assessment is now embedded, well-regarded and understood in the main, in comparison to the early days soon after its introduction (Minson, Brightwell and Long, 2012).

A utility model considering five variables: reliability, validity, educational impact, acceptability and cost, has been described for assessment methods ( van der Vleuten and Schuwirth, 2005; van der Vleuten, 2016; van der Vleuten, 1996). Each variable was weighted according to the importance attached by the user in a particular assessment context to denote compromise necessary in certain areas of assessment. This has been used to review START.

### Reliability

van der Vleuten *et al.* (2010) suggest structured and standardised instruments do not guarantee reliability and subjective evaluations are acceptable. Global ratings reduce inter-rater reliability but are offset by a larger gain in inter-station reliability. START’s trainee grading scheme would uphold that reliability. Global ratings are a more faithful reflection of expertise than a checklist ( van der Vleuten and Schuwirth, 2005).

Cronbach’s alpha as a measure of reliability are acceptable for an OSCE assessment of this nature. It will always be challenging for START to achieve high alpha values due to the homogeneity of the trainees undertaking the assessment in terms of knowledge and skill (as START is placed at the end of their training programme), the relatively small number of scenarios and the varying facets of clinical decision making and scenario thought processes being assessed; although these are all key skills for practicing as a consultant (which is the overarching construct), the scenarios cover a broad range of topics.

### Validity

In assessing the ‘does’ at the pinnacle of Miller’s pyramid, global rating, performance on rating scales and written narrative comments on positive and negative points on performance are appropriate ( Miller, 1990; van der Vleuten *et al.*, 2010). While usually applied to direct observations in-situ, using such formative models in an assessment is novel. As well as real-time prescribing, teaching and critical appraisal, START allows rehearsal of the professional conversation with a colleague. Some of the ‘doing’ scenarios are reported as more challenging. Some scenarios allow actual performance within the structured objective format allowing task-competency to be assessed.

### Educational impact

There is no doubt that assessment drives learning ( Schuwirth L, van der Vleuten C, 2004) and in that way more senior paediatric trainees make efforts to hone their critical appraisal skills and prescribing skills as well as consider the other aspects of the scenario domains. However the college does not advocate preparation for START as such and training itself should be enough. Now that the assessment is embedded, educational supervisors have more experience at supporting trainees through the aftermath of the assessment feedback and interpret the feedback into a useful Personal Development Plan for the penultimate year. This constructive alignment ( Biggs, 1996) maps their intra-assessment experience with a documented and evidenced outcome within their e-portfolio as they move into their penultimate training year. Much of the subject matter has been shown to be helpful not only for consultant working once appointed, but with the transition into the role, especially the consultant interview which may want to check out a trainee’s thinking on the way to appointability at that role ( Reece and Foard 2020, in press).

### Cost

No assessment is without resource implications. But cost can be offset for the organisation by careful budget management and finding value in assessing many trainees in one sitting. Multi-station assessments, like this one, are more efficient. Trainees paid separately to do START early on, but it is now offered as part of the cost of training, included in their annual training fee to the college.

### Acceptability

Assessment needs to be acceptable to both students and faculty. START has survived the first 6 years and, in that time, 13 diets without mutiny from either. That is not to say there have not been challenges. Some of which are discussed in the linked paper ( Reece and Foard 2020, in press). As one of the tools for determining progress and supporting learning in the workplace by giving direction, it has inherent value in the paediatric training assessment portfolio.

Much is made of the London-centric nature of the assessment, being held in the RCGP Examination Centre in London (which challenges the notion that it is ‘not an exam’ but a formative assessment). The logistics of needing to assess a large number of trainees from any number of 20 sub-specialties means that three concurrent circuits are run in two sessions over two days. The exception of this being the extra “half diet” (two circuits only over one day) undertaken in November 2017. The smaller numbers allowed us to move the assessment to a venue in the Midlands which demonstrates a level of flexibility and moved the assessment away from central London reducing travel logistics for some trainees.

## Conclusion

The RCPCH has successfully incepted, piloted and introduced a novel assessment for senior paediatric trainees towards the end of specialty training bringing externality at this stage of training. The formative nature of the assessment gives trainees areas of development to work on in their final year. The domains of the multi-scenario, OSCE-style assessment map to the domains of Good Medical Practice and map easily to the GMC’s Generic Professional Capabilities ( General Medical Council, 2017). As increasing numbers of trainees have taken the assessment, it has become embedded as a useful tool providing the trainees with feedback to help them develop themselves further in the final training year in preparation for consultant readiness, supporting their transition.

## Take Home Messages

• A novel mandatory, formative, multi-scenario, OSCE-style assessment has been successfully introduced in penultimate year of paediatric specialty training.
• Aspects of this assessment hold up well to a described utility model for assessment methods including reliability, validity, educational impact, acceptability and cost.

## Notes On Contributors

**Dr Ashley Reece** is a Consultant Paediatrician and Medical Educator. He has been involved in the Royal College of Paediatrics and Child Health examinations and assessments for 15 years and was the first Chair of the START Assessment Board between 2012 and 2016. He is currently the college’s Officer for Assessment. He successfully completed an MA in Medical Education in 2017.

**Lucy Foard** is a Psychometric Researcher at the Royal College of Paediatrics and Child Health. She has worked for the psychometric team within the College for 11 years, having previously held the roles of Psychometric Analyst and Psychometrician. She provides psychometric advice and guidance to other Royal Colleges and sat on the panel which developed guidelines for standard setting postgraduate examinations for the Academy of Medical Royal Colleges.

## Appendices

### Appendix 1 START Generic Benchmarking Grid

START Benchmark Standards

### Appendix 2 Exemplar of feedback sent to trainees following the assessment in 3 sample scenarios.

**Table:**

***Critical Appraisal Scenario*** You need to spend some time developing your critical appraisal skills. Although you showed understanding of the basics of reading a scientific paper, you did not demonstrate a structured approach to critical appraisal. I suggest you could do a critical appraisal course or get involved in your local journal club. These resources may help: https://www.cebma.org/resources-and-tools/what-is-critical-appraisal/ https://www.cebm.net/2014/06/critical-appraisal/ ***Safe Prescribing*** You completed the prescription chart accurately and legibly. The doses were correct and you reduced them in line with the known renal impairment in this patient as per the BNFC. You had good knowledge of the side effects of the medication and would counsel the parents on what to look out for. Your plan for monitoring drug levels ensured your safe approach to prescribing. Well done. ***Acute Scenario Based Discussion*** This was a tricky scenario of a teenager with anorexia presenting with cold extremities, low blood pressure and dehydration to the Emergency Department on a Friday afternoon. You realised the need for careful fluid resuscitation. It would be fine to involve your local PICU team for advice. You were aware of the NICE guidelines for anorexia nervosa, but not of the RCPsych Guidelines for Management of Really Sick Patients under 18 with Anorexia Nervosa. https://www.rcpsych.ac.uk/usefulresources/publications/collegereports/cr/cr168.aspx ***Handover*** You were able to prioritise the patients with the most urgent need in the handover sheet. You deployed your staff effectively taking account of the nursing shortages on the shift. This is clearly a situation you are familiar with. You paid attention to the child with a safeguarding need but were not distracted from the children with more acute medical issues. You needed a prompt to consider sending the FY1 doctor to arrange the skeletal survey for the infant with unexplained fracture so that could be done early in the shift, but you had a good plan to split the workload in this scenario where there were two potentially unwell and unstable children in asking the ST4 doctor to assess one potentially sick child while you dealt with the other one.

## Declarations

The lead author was the first START Executive Chair from the assessment’s inception in 2012 to 2016. This research was performed as part of a dissertation towards a Masters in Medical Education.
