# Supplementary material for: START – introducing a novel assessment of consultant readiness in Paediatrics: the entry not the exit
Source: MedEdPublish (2016). 2021 Sep 24;9:95. Originally published 2020 May 12. [Version 2] doi: 10.15694/mep.2020.000095.2 (PMC10697580; doi:10.15694/mep.2020.000095.2)
Supplement: Peer review history

# Reviewer report: Reviewer response for version 2

**DOI:** 10.21956/mep.20266.r31453

**Published:** 2021-10-07

**Authors:** Anita Samuel

This review has been migrated. The reviewer awarded 4 stars out of 5 This is an informative article on an effective form of assessment. Your clear descriptions and resources provided could help others who might be interested in implementing similar assessments in their contexts. It would be interesting to hear the consultants’ perspectives on this assessment style.

Reviewer Expertise:

NA

No decision status is available

# Reviewer report: Reviewer response for version 2

**DOI:** 10.21956/mep.20266.r31454

**Published:** 2021-10-02

**Authors:** Ken Masters

**Affiliations:** 1 Sultan Qaboos University

This review has been migrated. The reviewer awarded 4 stars out of 5 The authors have addressed my major concerns around Version 1 (The contextual background of the assessments, and moving Appendix 1 to a supplementary file). The context has made the impact of the work far easier to gauge, and having the supplementary file makes the material far more accessible. The paper is a good read, and is a useful contribution to the field.

Reviewer Expertise:

NA

No decision status is available

# Reviewer report: Reviewer response for version 1

**DOI:** 10.21956/mep.19003.r27259

**Published:** 2020-10-12

**Authors:** Ken Masters

**Affiliations:** 1 Sultan Qaboos University

This review has been migrated. The reviewer awarded 3 stars out of 5 An interesting paper describing the Royal College of Paediatrics and Child Health’s START assessment towards the end of specialist paediatric training.The paper begins by describing the idea that, rather than have a single summative assessment at the end of the training, there would be a formative assessment in the latter stages – allowing for a more comprehensive assessment and also having the advantages of formative assessments. The pilot proved successful, and the system was implemented.While the paper makes interesting reading, the assessment context does need to be described in some detail. International readers would see no mention of any other assessment, and may, therefore, believe that these specialists normally proceed through eight years of specialist training without a single assessment; the change now introduced is a single (albeit comprehensive) formative assessment in the latter half of their training. So, the authors need to give some details about assessment taken before and after this formative assessment, and any correspondence (or differences) between them.Other:For Appendix 1: I would recommend that this be included as a supplementary document, rather than a link. Many readers would be loath to click on a link that takes them off-site, and so would not access this material.I look forward to Version 2 of this paper in which the context, and, therefore rhe deeper implications, of this assessment are described.

Reviewer Expertise:

NA

No decision status is available

# Reviewer report: Reviewer response for version 1

**DOI:** 10.21956/mep.19003.r27260

**Published:** 2020-05-13

**Authors:** Dujeepa D. Samarasekera

**Affiliations:** 1 National University of Singapore

This review has been migrated. The reviewer awarded 2 stars out of 5 Interesting article which describes an OSCE style assessment at the end or near the end of the training for trainee's readiness for tenure as a consultant. It would have been better if the authors could elaborate on how this particular test would add value to the multiple evaluations that the resident has gone through earlier in the program. The resident at the very end of his or her program should be at a higher level of "Does" or "Is" level of Millers/modified Miller's pyramid, which I understand that the current portfolio comprising all the supervisors' reports and workplace-based assessments capture. Is there any area that is missed in the current assessments? Are there any significant gaps this particular assessment is trying to capture? Or is this part of the medical education dissertation and a pilot? Planning assessment is very important and we try as much as possible to reduce the burden for our residents and faculty/administrators. Therefore, it is important to understand the construct of this tool in the overall performance evaluation of the resident. The rest of the paper is good reading. Thank you.

Reviewer Expertise:

NA

No decision status is available
